# A comparative analysis of non-invasive prenatal testing in Ontario and Quebec: the role of governing style in health technology innovation & adoption

**DOI:** 10.1186/s12913-023-09245-6

**Published:** 2023-03-09

**Authors:** Lena Saleh, Gillian Parker, Michael Stevenson, Fiona A. Miller

**Affiliations:** 1 Institute of Health Policy, Management and Evaluation, University of Toronto, 155 College St 4Th Floor, Toronto, ON M5T 3M6 Canada; 2 School of Public Health and Health Systems, University of Waterloo, 200 University Avenue West, Waterloo, ON N2L 3G1 Canada

**Keywords:** Comparative research, Health politics, Health technology, Regulation, Governance, Non-invasive prenatal testing, NIPT

## Abstract

**Background:**

While processes of adoption and the impacts of various health technologies have been extensively studied by health services and policy researchers, the influence of policy makers’ governing styles on these processes have been largely neglected. Through a comparative analysis of non-invasive prenatal testing (NIPT) in the Canadian provinces of Ontario and Quebec, this article examines how decisions about this technology were shaped by contrasting political ideologies, resulting in vastly different innovation and adoption strategies and outcomes.

**Methods:**

A comparative qualitative investigation comprising of a document analysis followed by semi-structured interviews with key informants. Interview participants were researchers, clinicians, and private sector medical laboratory employees based in Ontario and Quebec, Canada. Interviews were conducted both in person and virtually– owing partly to the COVID-19 pandemic – to garner perspectives regarding the adoption and innovation processes surrounding non-invasive prenatal testing in both provinces. All interviews were recorded and transcribed verbatim and data were analyzed using thematic analysis.

**Results:**

Through an analysis of 21 in-depth interview transcripts and key documents, the research team identified three central themes: 1) health officials in each province demonstrated a unique approach to using the existing scholarly literature on NIPT; 2) each provincial government demonstrated its own preference for service delivery, with Ontario preferring private and Quebec preferring public; and finally, 3) both Ontario and Quebec’s strategies to NIPT adoption and innovation was contextualized within each province’s unique financial positioning and concerns. These findings illustrate how both Quebec’s nationalist focus and use of industrial policy and Ontario’s ‘New Public Management’ style had implications for how this emerging healthcare technology was made available within each province’s publicly-financed health system.

**Conclusions:**

Our study reveals how these governments’ differing approaches to using data and research, public versus private service delivery, and financial goals and concerns resulted in distinct testing technologies, access, and timelines for NIPT adoption. Our analysis demonstrates the need for health policy researchers, policy makers, and others to move beyond analyses solely considering clinical and health economic evidence to understand the impact of political ideologies and governing styles.

## Background

Scholars have begun probing the connections between neoliberal political ideologies and forms of territorial and institutional reform within healthcare systems. Arguments have been made regarding the reorganization and restructuring of power by neoliberal ideologies [1]; its influence on the operations of health systems [2]; the impact of varied levels of privatization [3] and its role in strengthening the public health complex [4]. Additionally, a body of literature has examined the various impacts of these reforms on healthcare practitioners’ capacities to provide quality care to patients [5, 6]. Less attention has been given to how governing styles influence the processes through which new health technologies, such as molecular genetic tests, are developed and adopted into publicly-financed health systems. (By ‘governing style, we mean the set of processes and assumptions that work to inform how policy decisions are reached.) While the socio-political and economic impacts of genetic testing have been analyzed in the literature [7–9], few studies have examined relationships between governing styles and decisions to adopt these new platforms. In this paper, we address these gaps, arguing that governing styles have a significant influence the processes through which health technologies come to be adopted and delivered by publicly-financed health systems.

One relatively new genetic test is cell-free fetal DNA (cffDNA) screening, also known as non-invasive prenatal testing (NIPT). In 1997, Lo and colleagues [10] determined that fragments of fetal DNA are present in maternal blood. The commercialization of NIPT for atypical fetal chromosome counts began once researchers could analyze cffDNA from maternal blood in high volumes [11–14]. Within four years of the first tests entering the market in 2011, NIPT was being used in 60 countries around the world [15, 16] to screen for trisomies 21, 18, and 13.

While NIPT is currently available for purchase by all pregnant women in Canada, not every province covers its costs under their health insurance schemes. Canada’s two most populous provinces, Ontario and Quebec, offer publicly-financed NIPT for high-risk pregnancies (e.g., maternal age above 40 years, the existence of trisomies 21, 18, and 13 within the family, or a twin pregnancy), though each has chosen a different pathway to innovation and adoption.

We argue that the approach to adoption of NIPT in Ontario was influenced by the province’s ‘New Public Management’ (NPM) governing style. NPM can be understood as an ‘administrative doctrine’ seeking to slow down or reverse government growth in terms of public spending and staffing, while expressing a preference towards privatization [17, 18]. NPM is part of neoliberal trends that emerged in the 1980s. Neoliberalism constitutes a set of socio-economic and political assumptions, grounded in classic ‘laissez-faire’ economics, that maintain economic growth and development, and are contingent upon the following: reducing public deficits; broadening the tax base, reducing corporate taxes and domestic sector subsidies, and privatizing government-controlled industries [19]. Within the context of healthcare, this governing style has manifested as forms of ‘contracting out’ services to private entities, to lower government expenditures [3, 20, 21]. This decision can lead to favouring efficiency over transparency as private entities are not required to publicly share proprietary information [22]. Additionally, decisions to cut public spending overall led to decreased healthcare spending. In Ontario, this translated into, “overcrowded emergency rooms, understaffed and underequipped cancer and cardiac treatment centers, and shortages of new high-technology diagnostic equipment …” [23].

Conversely, the Quebec approach to the adoption of NIPT can be understood as a form of economic and industrial ‘nationalism,’ which sought to promote a ‘Quebec-first’ stance, encouraging investment in Quebec through the deployment of a directed industrial policy. Quebec’s approach to adoption of NIPT reflects a resurgence of interest in industrial policy in both Canada and globally after the global financial crisis (against a background of longstanding, if hidden, developmental state activity) [24, 25]. Occurring within the majority-French ‘nation within a state’ that is Quebec, this approach to adoption of NIPT confirms the insights of existing studies on sub-national politics and stateless nations. Scholars highlight the importance of public policy for maintaining and strengthening forms of sub-nationalist mobilization [26], contributing to a ‘sense of difference’ and institutional distinctiveness through state ‘ownership’ of public policies that are designed to respond to the needs of the nation [27].

Through a comparative analysis of the distinctive approaches to NIPT in Ontario and Quebec, this article explores how the technology’s development, adoption, and delivery was shaped by the governing styles in each province.

## Methods

This paper presents a comparative, qualitative investigation of key documents blended with semi-structured interviews. This study was granted ethics approval by the Office of Research Ethics at the University of Toronto (Protocol #00,033,786).

We conducted twenty-one interviews with physicians, geneticists, and representatives from both regulatory bodies and pharmaceutical companies between 2019–21. Recognizing the existence of professional relationship networks amongst individuals working in this space, a snowball sampling approach was used to recruit participants, primarily within the Canadian provinces of Ontario and Quebec [28].

Amongst the participants, six were geneticists/obstetricians based in Ontario, seven were geneticists/obstetricians based in Quebec, five worked with national regulatory bodies, two were representatives from an international pharmaceutical company, and one was a researcher/clinician based in British Columbia. Given the limited scope of the study, the research team concluded that the collected interviews, when supplemented with additional sources (i.e., government publications, journal articles, etc.), offered a sufficient base to answer research questions [29].

The interviews were conducted both in-person and virtually by three research team members. All participants were given a set of interview questions in advance, and all participants provided written informed consent to participate. Interview data was later anonymized by the research team. Interviews ranged from approximately forty-minutes to one hour, were recorded, and transcribed verbatim. All interviews were conducted in English.

Transcripts were analyzed using a thematic analysis approach [30]. Two researchers successively read and coded the transcripts, documenting codes they perceived to be relevant to the research question. Both researchers then actively discussed their interpretations of the data and identified themes. To ensure consistency, a third team member independently coded a sample of interview transcripts, which were then compared against the first team members’ coding. From the perspective of the authors, this collaborative and conversational style of coding allowed for author reflexivity by acknowledging the role of subjectivity in data analysis [29].

## Results

**Fig. 1:**
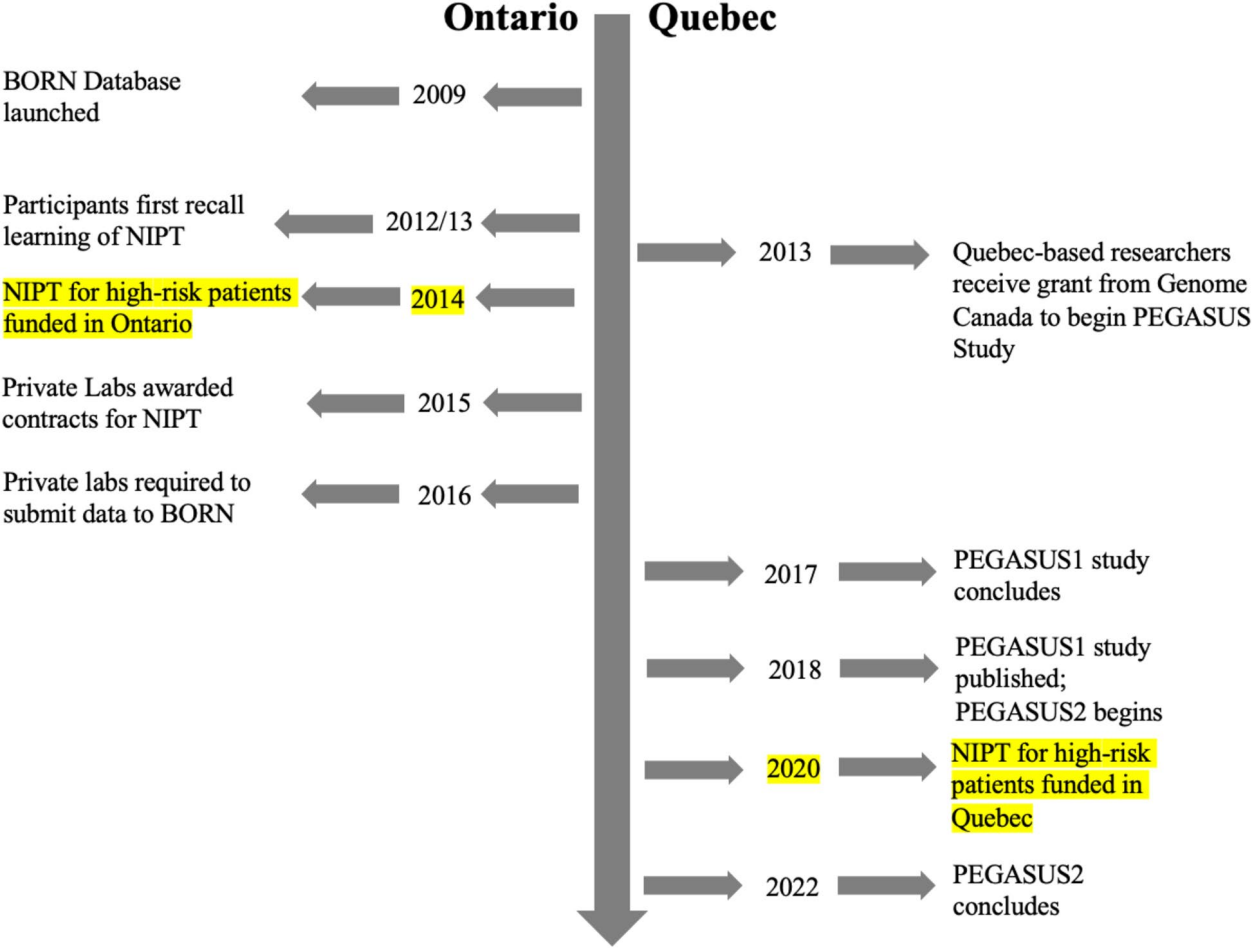
NIPT Adoption Timeline

Below we present a case summary of Quebec’s and Ontario NIPT adoption strategies, followed by themes identified in the semi-structured interviews, illustrating how the influence of the political ideologies of these two provinces resulted in vastly different approaches and outcomes. See Fig. 1. for the NIPT adoption timeline in Ontario and Quebec.

### Ontario’s road to NIPT

NIPT was first available to pregnant women in Ontario in 2012 as an out-of-pocket expenditure. Shortly after, an expert committee recommended that while NIPT would financially burden the province, the overall reductions in invasive procedures (e.g., amniocentesis) would still produce savings [31]. Thus, in 2014, the Ontario government opted to include NIPT for high-risk pregnancies under its publicly-financed health insurance. Initially, samples were sent out-of-country for analysis, but in 2014 the province decided to patriate the test.

As part of this, the Ontario government decided to contract out the service through a (quasi) open competition amongst both public and private laboratories, eventually awarding the contract to two private laboratories: *LifeLabs* and *Dynacare.* Each laboratory had already forged partnerships with American test manufacturers (*Airosa* and *Natera* to use their *Harmony* and *Panorama* tests, respectively) prior to competing for the licenses required to complete testing.

Prior to this competition, it had been “at least 17 years” since a private laboratory had been awarded a license to conduct publicly-financed tests [32]. This barrier, however, was circumvented by *LifeLabs* and *Dynacare* through their respective acquisitions of existing licensed laboratories.

### Quebec’s road to NIPT

Unlike in Ontario, the Quebec government developed and delivered its own ‘homebrew’ NIPT, and it is the only publicly-financed test in the province. The development of this test was funded by a research grant from *Genome Canada*, a federally-funded non-profit supporting the development and commercialization of genomic-based technologies. This study, acronymized as PEGASUS, was undertaken between 2013 and 2017 and had two objectives: to validate the performance of existing NIPTs; and, to develop a Quebec-made NIPT for use in public laboratories that could match commercial tests’ efficacies.

When PEGASUS was launched in 2013, there were already four American corporations dominating the NIPT market: *Sequenon*, *Verinata*, *Ariosa*, and *Natera*. Each not only reported similar performance data on their tests but were also all involved in litigation regarding the protection of their testing methodologies [14]. Despite this, there remained a skepticism and hesitancy amongst Quebec researchers and policy makers to adopt this technology as early studies were industry-funded. In 2018, PEGASUS concluded and published its results.

In 2018, the original PEGASUS study was extended for several more years, becoming PEGASUS2 (2018–22). PEGASUS2 had several objectives, including locating further avenues for cost reduction, examining the possibility of expanding the test to include other anomalies, and the development of web-based tools to increase patient access to information. PEGASUS2 was already underway when the assay developed in PEGASUS was integrated into Quebec’s prenatal screening program in early 2020.

### Thematic analysis

We identified three themes that illustrate the influence of policy makers’ governing styles on these provincial approaches to NIPT: 1) approaches to using existing research on NIPT; 2) disposition toward public versus private service delivery; and 3) financial implications and concerns.

#### Theme one: approaches to using existing data and research on NIPT

##### Ontario

By 2016, both the *Harmony* and *Panorama* NIPTs had undergone large-scale clinical trials in the United States [33–35]. These studies, however, were funded by industry. While this fueled decisions to independently evaluate NIPT platforms in Quebec for fear of bias within the clinical trials, Ontario’s NPM-inspired government desired to promote efficiency, making the test available as quickly and cheaply as possible, overriding concerns about biases. Indeed, one Ontario participant explained that while most of the research on NIPT was funded by venture capital, the studies still met the criteria set by the National Institutes of Health. On this basis, this same participant described PEGASUS as a “useless study” that only concluded the test “works like it worked with everyone else” [P7].

Ontario, according to participants, was willing to accept the validity of existing data, but did take steps to include a quality assurance mechanism. NIPT results in Ontario were incorporated into the province’s existing birth outcomes registry, known as Better Outcomes Registry and Network (BORN). BORN is Ontario’s official registry for health administrative data related to maternal, infant and child health. The government asked vendors to share data, allowing for retrospective analysis of commercial test performance by BORN. Approximately two years after NIPT was financed, *Dynacare and LifeLabs* were required to upload the data from all patients into BORN’s database. Data from NIPTs in Ontario, therefore, has been collected since January 2016, allowing researchers and clinicians to generate a longitudinal dataset to better assess test performance and usage. One Ontario clinician described this arrangement as “very reassuring,” describing it as a “good QA [quality assurance] system” [P1].

##### Quebec

A participant observed that the “philosophy” of the Quebec government regarding laboratory medicine was that “they don’t want any private involvement” [P4]. This ‘philosophy’ also explained why Quebec was hesitant to accept existing published data on commercially available NIPT because of its connection to industry [36, 37].

While data suggested similar performance of the four tests [14, 35], the producers of these diagnostics funded initial validation studies. Moreover, due to a regulatory loophole, these commercial assays had not been independently reviewed by the United States Food and Drug Administration or Health Canada [14, 37–40]. Participants explained that the “main push” for PEGASUS was that existing data was industry-funded [P2]. As one Quebec participant noted, the commercial tests had been “developed in secrecy and were like black boxes” and either “you believed in the results, or you did not” [P3].

#### Theme two: public versus private service delivery

##### Ontario

In Ontario, the decision to utilize existing commercial tests and private laboratories resulted in an expedited adoption and delivery of NIPT. One Ontario clinician recalled how quickly the test was introduced into the Ontario healthcare system, noting that “all of a sudden it was just here” [P1]. They continued, explaining that manufacturers themselves were reaching out directly to clinicians, presenting themselves as “partners”.

Ontario also decided that its adoption of NIPT should be an “open process” available to all licensed labs. One participant from the Ministry of Health explained that NIPT was part of a larger project to adopt several new genetic tests. NIPT was part of an open competition, they continued, through which all licensed labs, public or private, in Ontario could apply. The province’s decision to award contracts, further, was based on a “multidimensional evaluation of the applications” that considered cost, technical aspects, and expertise of the labs applying [P11].

##### Quebec

Unlike in Ontario, the inclusion of private entities was not presented as an option in Quebec’s NIPT adoption and delivery strategy. A Quebec-based clinician-researcher observed that the government had a “real objective” of keeping the test in the public system because it would allow for reduced costs and guarantee their access to the health data generated. If a private vendor had control, the healthcare system would be beholden to them, the participant explained [P4].

Another Quebec-based researcher discussed the province’s desire for control and independence from private entities. The participant stressed how, given the large volume of NIPTs that would be conducted, the province would want “control over how it’s done and not be dependent on a lab” that could possibly close or change its protocols [P2]. Thus, while Ontario used its BORN registry to assess NIPT’s quality and accuracy, Quebec opted for a more ‘hands on’ delivery style. By offering the test through state-owned laboratories, the province would retain control of all aspects of the process.

#### Theme three: financial implications, and concerns

##### Ontario

As mentioned above, NPM holds that increased governmental cooperation with private entities stands to lower public expenditures. In Ontario, this logic was deployed to justify increased outsourcing to private labs. The financial implications of these decisions, however, are incredibly difficult to assess and raise questions regarding the possibility increased spending and fiscal mismanagement due to a lack of transparency and disclosure. Unlike public entities, private labs are not required to release financial information relating to their profit margins. To do so, according to one participant from the private laboratory sector, would be “economically damaging” [P5]. Similarly, a researcher involved in the publication of a commissioned report on laboratory testing in Ontario lamented that as they worked to produce the report, they only had “the roundest numbers” as private entities’ financial information is “not disclosed to anybody” [P6].

To this end, an exact accounting of the costs associated with NIPT testing in Ontario is difficult (if not impossible) to achieve because the information regarding the price paid per test by private labs versus the price subsequently billed to the province remains undisclosed. For this reason, the single largest recommendation of the 2015 *Laboratory Services Expert Panel Report for Ontario* was that Ontario should renegotiate contracts with labs. “As part of the negotiation, the Ministry [of Health] should clearly stipulate upfront the minimum price discount it must obtain or conversely, the maximum price it would be willing to pay.” [32]. The report suggests including “audits” to “provide assurance to…taxpayers that value for money is being achieved…” [32].

Prior to the province’s call for bids for the NIPT contract, private labs had never received a licence to conduct genetic testing. Indeed, the 2015 Report recognized the possibility for fiscal mismanagement, accusing the province of failing to produce a “strategic plan” for addressing the governance, funding, and coordination” of NIPT [32]. Thus, while the increased use of private labs was sought out to reduce expenditures, the province’s sole clear achievement appears to be greater fiscal opacity vis-à-vis the spending of public funds.

##### Quebec

Quebec’s approach to NIPT, participants suggested, had significant financial motivations. One participant explained that the province was partly motivated by economic concerns [P4]. Quebec sought to maintain low lab costs and actively worked to guarantee that the knowledge and resources necessary to maintain this remained within the province. This underpinned the Quebec’s commitment to having public hospital labs perform as much of the testing workload as possible. A Quebec-based geneticist characterized these decisions as a “political value” in Quebec. The logic, they explained, was that if the health system was to devote significant resources to something, those resources ought to stay within Quebec. “If we build it locally, then people have jobs,” they observed, “all the money that’s being spent doing these tests [i.e., NIPT] stays in the economy” [P2].

This strategy was critiqued by some participants. Quebec spent $10 million CAD, lost time reproducing data that already existed, and as one Ontario physician/researcher [P7] argued, permitted delays in making NIPT available to high-risk pregnant women in Quebec until 2020.

## Discussion

Policy decisions regarding the development, adoption, and delivery of a health technology are often understood as a technical exercise for governments. Using the example of NIPT in Ontario and Quebec, this study explored the argument that these processes are also political. Specifically, that the political ideologies of governments shape health development, adoption, and delivery are approached by publicly-financed healthcare systems. Following public policy researchers Geva-May and Maslove [40], we illustrate that “health technology moves along the fault lines of other politics.”

In Canada, the delivery of health services is primarily the responsibility of the provinces, and each has responded uniquely to NPM-inspired political pressures [23]. NPM-style reforms across Canada were triggered by the political struggles between the federal and provincial governments and other health system stakeholders arising in the 1990s [41]. Scholars argue that Ontario and Alberta have adopted NPM “the most fully” [21], while Quebec has adopted it “the least” [42]. NPM rhetoric within Canadian healthcare, thus, must be understood with reference to each province’s political context and climate.

Pressures to increase for-profit entities’ participation have long been a political issue in Ontario. In the 1990s, Ontario’s government sought to slash healthcare spending [43], ultimately cutting $800 million CAD from hospital budgets, creating system-wide issues and bottlenecks. These cuts set the stage for private entities’ entrance as a means of addressing the issues of access, equity, and quality of care. Ontario introduced a system of ‘contract bidding’ that sought to maintain/promote reductions in spending, while ensuring patient access to basic services. These competitive bidding processes have been maintained by subsequent governments, despite non-profit entities’ criticisms that the private sector is capable of underbidding [23].

Our study of the case of NIPT adoption in Ontario demonstrates this political valuation. Ontario’s decision to partner with *Dynacare* and *LifeLabs* by awarding them exclusive contracts to perform the testing not only evidenced the government’s desire to slow down and reduce government growth and spending, but also its comfort with private entities’ involvement in the health system [20]. Through these contracts, Ontario’s government sought to make use of existing infrastructure (i.e., private labs) as a quick, low-cost means to provide a service. But what have been the consequences of this political decision? While it is still too early to fully assess the effectiveness of Ontario’s NIPT adoption and delivery strategy, there are reasons to believe that greater efficiency and cost-savings may not be realized.

First, private entities’ participation raises concerns about financial transparency. The process through which Ontario awarded lab contracts appears to be something of a ‘black box,’ a concern given the democratic ideal of public oversight vis-à-vis government spending. Our participants make little to no mention of public participation in the ‘open process’ through which the NIPT contracts were awarded, aligning with the observations of critics who note that the awarding of contracts through NPM-style reforms is often exclusionary, lacking stakeholder involvement [44].

Second, skepticism of NPM’s promise that implementing business and private sector ideas into public services would reduce costs and inefficiencies is warranted. Chief amongst these ideas is the promotion of competition [17]. NPM holds that the ‘trust’ placed in civil servants to oversee the management and operation of public services and organizations is misplaced because inefficiencies are continually funded [45]. Introducing elements of competition, through contracting out, is presented as a solution to these inefficiencies [46].

NIPT adoption in Ontario, however, appears to serve as a counterexample. As *LifeLabs* and *Dynacare* enjoy their exclusive licenses, other labs cannot offer NIPT. Consequently, these two labs capture all patient-pay testing in the province, allowing them to charge higher fees for the service. Further, private entities are not subject to routine audits, meaning *LifeLabs* and *Dynacare* are not required to disclose data pertaining to their profit margins. Thus, it is unclear whether outsourcing of this nature produces cost-savings.

Importantly, our findings parallel the work of scholars in other geographic contexts [46]. In Britain, Simonet [47] argues that there is “little evidence” suggesting that contracting out led to cost reductions, and in fact, may have contributed to worsening health outcomes for patients. More than twenty-five years of NPM experimentation has not produced greater accountability or reduced fraud because complexity has made the development of adequate controls challenging [47].

In Quebec, our participants’ insights reflect its unique positioning amongst the provinces as ‘a nation within a state’. This ‘exceptionalism’ manifests politically as Quebec’s occasional refusal to participate in federally initiated programmes [48]. Consequently, Quebeckers have, at times, been denied access to health, education, and welfare provisions available elsewhere in Canada [27]. Quebec’s national identity is often expressed in the institutions and policies of the Quebec state. Connections between nation and state have justified governmental intervention in social, economic, and cultural policies. It is not enough that Quebeckers have access to the same social welfare provisions as other Canadians, but that these policies were “developed, financed, and provided by the government of Quebec” [27].

Canadian provinces, therefore, have a history of pragmatically deploying public policy to support industrial policies reflective of current “political realities” [24]. Quebec’s willingness to intervene to preserve its national identity and maintain control over particular economic sectors is a well-documented example of this phenomenon. Indeed, Quebec’s industrial policy has long sought to both support the province’s economy, reinforcing a ‘Quebec-first’ stance. In pursuit of this, the Quebec government has stressed the function of provincially-owned corporations [49].

Our analysis of NIPT in Quebec exists within this political context. PEGASUS can be understood as Quebec’s desire to support itself and avoid reliance on others/outsiders. First, the study was premised on the need to validate the data generated *outside* Quebec/by others. Second, Quebec created its ‘homebrew’ test to weaken its ties to and reliance on tests produced by industry. By weakening these ties vis-à-vis NIPT, Quebec’s strategy worked to strengthen and invest in state-owned institutions. While private labs do offer NIPT as a fee-for-service, the only provincially-financed test is the ‘homebrew.’

Two points emerge: first, the government of Quebec sought to reinforce its caretaker role vis-à-vis the health of Quebeckers, promoting its exceptionalism; second, the government’s insistence on offering of NIPT through public labs paints a clear policy picture, where the state prioritized itself and its objective of control by investing in its institutions (i.e., job creation and keeping money within Quebec), without sacrificing state power to private entities.

But what are the consequences of this strategy? We can speculate that the use of the ‘homebrew’ test is a low-cost option for the province. Quebec is largely freed from navigating costly contracts with private labs or litigious commercial manufacturers. Potentially most seriously is the criticism that has been levelled against Quebec’s nationalist strategy. PEGASUS led to delays in service provision for pregnant women in Quebec, as the publicly-financed NIPT (using the ‘homebrew’ test) was not made available until 2020 (more than five years after it was funded in Ontario). This delay raises important questions for further research to explore regarding the province’s prioritization of nationalist goals over the delivery of certain health services.

Our analysis of NIPT in Quebec also confirms the insights of others regarding the connections between national identity and healthcare. In Cuba, political scientist Johnson [50] argues that “health” is conceived as an expression of its commitment to socialism becoming “indispensable to that country’s sense of nation and source of legitimacy.” “Health” in Cuba works to “defend […] against competing [socio-political] visions at home and abroad” [50]. In Britain, public health researcher Cowan [51] similarly discusses the “backlash” from activists concerning the “international policy trend to contract healthcare out to (often multinational) private companies.” Health activists, Cowan argues [51] frame the National Health Service as the “beacon of equality,” using it to justify British distinctiveness. In Austria, political scientist Metzler [50] argues that the enshrinement of a particular “set of visions” into legislation and implemented within the public healthcare system created a unified vision of what prenatal care ought to contain. The pre-existence of this vision, she [52] argues allowed NIPT to spread easily within Austria because it could “travel in and through the moral and material grounds of an extant imaginary of prenatal testing while also reifying it.” While most Canadians likely have strong feelings about the country’s public provision of health services vis-à-vis other countries, the example of NIPT in Quebec works to connect the province’s unique identity and feelings of difference to healthcare.

### Study strengths and limitations

This study seeks to be an early contribution to a health policy literature exploring the connections between political ideologies of governance and health technology adoption and innovation. Out study has several strengths. First, our research participants included individuals working within both the private and public sectors, allowing for better comparison between the two spaces. Second, we supplement and cross-reference these interviews with information taken from key government reports and publications, thereby adding additional context to the opinions and experiences of participants. And third, our study raises important questions regarding the relationship between the achievement of political and economic goals and women’s ability to access reproductive information. Despite these strengths, more research is needed to compare the longer-term effectiveness of these strategies. Such work, however, will be challenging given industry’s penchant for privacy and unwillingness to release financial information/records. Additionally, a subsequent study able to include French-language participants could add greater nuance to perspectives on NIPT in Quebec, particularly regarding the province’s unique positioning within Canadian politics. Further, while our comparative work has focused on the cases of Quebec and Ontario, provincial control over healthcare in Canada creates fertile ground for further comparative work on health technology adoption across provinces.

## Conclusion

The utility of new health technologies is not the sole factor influencing the policy decisions impacting their development, adoption, and provision. Our analysis of NIPT in Ontario and Quebec is an exemplar to advance our understanding of the complex interconnections amongst various socio-political realities and the (state-)adoption of novel health technologies. Our analysis demonstrates the need for health policy researchers, policy makers, and others to move beyond analyses exclusively considering clinical and health economic evidence to include consideration of the ideological underpinnings of governments.

## Data Availability

The datasets supporting the conclusions of this article are included within the article.

## Abbreviations

BORN: Better Outcomes Registry and Network
cffDNA: Cell-free fetal DNA
NIPT: Non-invasive prenatal testing
NPM: New public management
PEGASUS: Personalized Genomics for Prenatal Aneuploidy Screening Using Maternal Blood
